# Supplementary material for: Real word evidence on rituximab utilization: Combining administrative and hospital-pharmacy data
Source: PLoS One. 2020 Mar 12;15(3):e0229973. doi: 10.1371/journal.pone.0229973 (PMC7067445; doi:10.1371/journal.pone.0229973)
Supplement: S1 Table — The table shows the total number of hospitalizations due to adverse infectious events recorded up to one year from first rituximab administration (events were also subdivided by Non-Hodgkin Lymphoma (NHL) and Chronic Lymphocytic Leukemia (CLL)). ICD-9CM codes about infectious events were reported. (DOCX) [file pone.0229973.s001.docx]

**Table S1. Hospitalizations due to adverse infectious events recorded up to one year from the first rituximab administration ***

|  |  | **Indications** | | |
| --- | --- | --- | --- | --- |
| **Diagnosis** | **ICD9CM** | **NHL  (n=264)** | **CLL (n=47)** | **Total**  **(n=311)** |
| Patients with ≥1 adverse infectious event |  | 7(2.6) | 2 (4.2) | 9 (2.9) |
| Women (%) |  | 3 (12) | 0 | 3 (1,5) |
| Admitted to University Hospital of Siena (n) |  | 7(2.6) | 2 (4.2) | 9 (2.9) |
| **Sepsis** |  | 6 (2.3) | 1(2.1) | 7(2.2) |
| Other staphylococcal septicemia | *038.19* | 0 | 1(2.1) | 1(0.3) |
| Septicemia due to other gram-negative organisms | *038.40* | 1(0.4) | 0 | 1(0.3) |
| Septicemia due to E.Coli | *038.42* | 1(0.4) | 0 | 1(0.3) |
| Other septicemia due to other gram negative organisms | *038.49* | 1(0.4) | 0 | 1(0.3) |
| Systemic inflammatory response syndrome due to infectious process without organ dysfunction | *995.91* | 2(0.7) | 0 | 1(0.3) |
| Septic shock | *785.52* | 1(0.4) | 0 | 1(0.3) |
| **Adenovirus infection** |  | 1 (0.4) | 1 (2,1) | 2 (0,6) |
| Herpes simplex | *054.** | 0 | 1(2.1) | 1(0.3) |
| Herpes zoster | *053.** | 1(0.4) | 0 | 1(0.3) |

NHL: Non-Hodgkin Lymphoma; CLL: Chronic Lymphocytic Leukemia

*only primary diagnosis were considered
