# Supplementary material for: Real word evidence on rituximab utilization: Combining administrative and hospital-pharmacy data
Source: PLoS One. 2020 Mar 12;15(3):e0229973. doi: 10.1371/journal.pone.0229973 (PMC7067445; doi:10.1371/journal.pone.0229973)
Supplement: S3 Table — The table shows the time-to-onset of adverse infectious events occurred up to one year from the first rituximab administration (only primary positions of hospital discharge records). Meantime, median time, as the range days of the time-to-onset were reported. (DOCX) [file pone.0229973.s003.docx]

**Table S3. Time-to-onset of adverse infectious events that occurred up to one year from the first rituximab administration ***

|  | **Cases** | **Mean time (days)** | **Median time (days)** | **Range (days)** | **Time in months (n)** | | |
| --- | --- | --- | --- | --- | --- | --- | --- |
|  |  |  |  |  | < 3 months | From 3 to 6 months | > 6 months |
| Sepsis | 7 | 107,85 | 92 | (23-264) | 3 | 3 | 1 |
| Adenovirus infection | 2 | 52,5 | 52,5 | (22-83) | 2 | 0 | 0 |

*only primary diagnosis were considered
